# Late relapse of IgM nephropathy‐associated nephrotic syndrome after repeated administration of immune checkpoint inhibitor against pulmonary adenocarcinoma

**DOI:** 10.1002/ccr3.3903

**Published:** 2021-03-02

**Authors:** Kentaro Odani, Mitsuhiro Tachibana, Fumiaki Nogaki, Yutaka Tsutsumi

**Affiliations:** ^1^ Department of General Medicine (junior resident) Shimada Municipal Hospital Shimada Japan; ^2^ Department of Diagnostic Pathology Shimada Municipal Hospital Shimada Japan; ^3^ Department of Nephrology Shimada Municipal Hospital Shimada Japan; ^4^ Diagnostic Pathology Clinic Nagoya Japan; ^5^ Department of Diagnostic Pathology Kyoto University Hospital Kyoto Japan

**Keywords:** acute pancreatitis, IgM nephropathy, immune checkpoint inhibitor, immune‐related adverse events, interstitial pneumonia, pulmonary adenocarcinoma

## Abstract

ICPIs were effective for primary and metastatic foci of lung adenocarcinoma, but their repeated use provoked a late relapse of IgM nephropathy and lethal lesions in pancreas and lung. ICPIs should be used carefully in cases of immune‐related disease.

## Graphical abstract

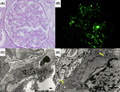

## 1 INTRODUCTION

A man in his 60s suffering nephrotic syndrome with IgM nephropathy seven years earlier received immune checkpoint inhibitors (ICPIs) for metastasizing pulmonary adenocarcinoma four years later. Sixty‐two weeks after 21 ICPI injections, adenocarcinoma vanished but nephrotic syndrome relapsed. As late‐onset immune‐related adverse events, acute pancreatitis followed and usual interstitial pneumonia exacerbated.

Immune checkpoint inhibitors (ICPIs) have increasingly attracted medical oncologists’ attention as the target malignant diseases of ICPIs have been expanded considerably. ICPIs activate T‐lymphocytes to attack tumor cells, but these activated T‐lymphocytes may also injure normal cells. Immune activation by ICPIs provokes immunoglobulin production and excessive release of a variety of cytokines, including interleukin‐17, ^1^ which may result in immune‐related adverse events (irAEs).

In most clinical trials of ICPIs, patients with immune‐related disorders were excluded from study. In fact, package leaflets of ICPIs seldom describe the disadvantage for patients with pre‐existing immune‐related disorders. The frequency of irAEs caused by ICPIs may increase particularly in patients with a poorly controlled immune‐related disorder or in those who manifest organ injury. ^2^ Reportedly, ICPIs provoked the relapse of the primary disease in 75% of patients with immune‐related disorder. ^3^

IgM nephropathy manifesting as nephrotic syndrome was first described in 1978. ^4^ Immunofluorescence study has revealed deposition of IgM in the mesangial matrix, and ultrastructurally, the deposit shows low electron density. IgM nephropathy‐related nephrotic syndrome often affects adults and is commonly steroid resistant. ^5^ The distinction between minimal change nephrotic syndrome and focal segmental glomerulosclerosis has long been debated. ^6^, ^7^ At present, the disease entity of IgM nephropathy remains to be established.

We report herein an autopsy case of a Japanese adult man presenting with late relapse of IgM nephropathy‐associated nephrotic syndrome after repeated administration of an ICPI, pembrolizumab (Keytruda^®^), against pulmonary adenocarcinoma with systemic metastasis. During the late clinical course, irAEs occurred in the pancreas and lung. The mechanisms of irAEs, particularly those of late‐onset irAEs, are discussed from the viewpoint of pathology.

## 2 CASE PRESENTATION

**FIGURE 1:**
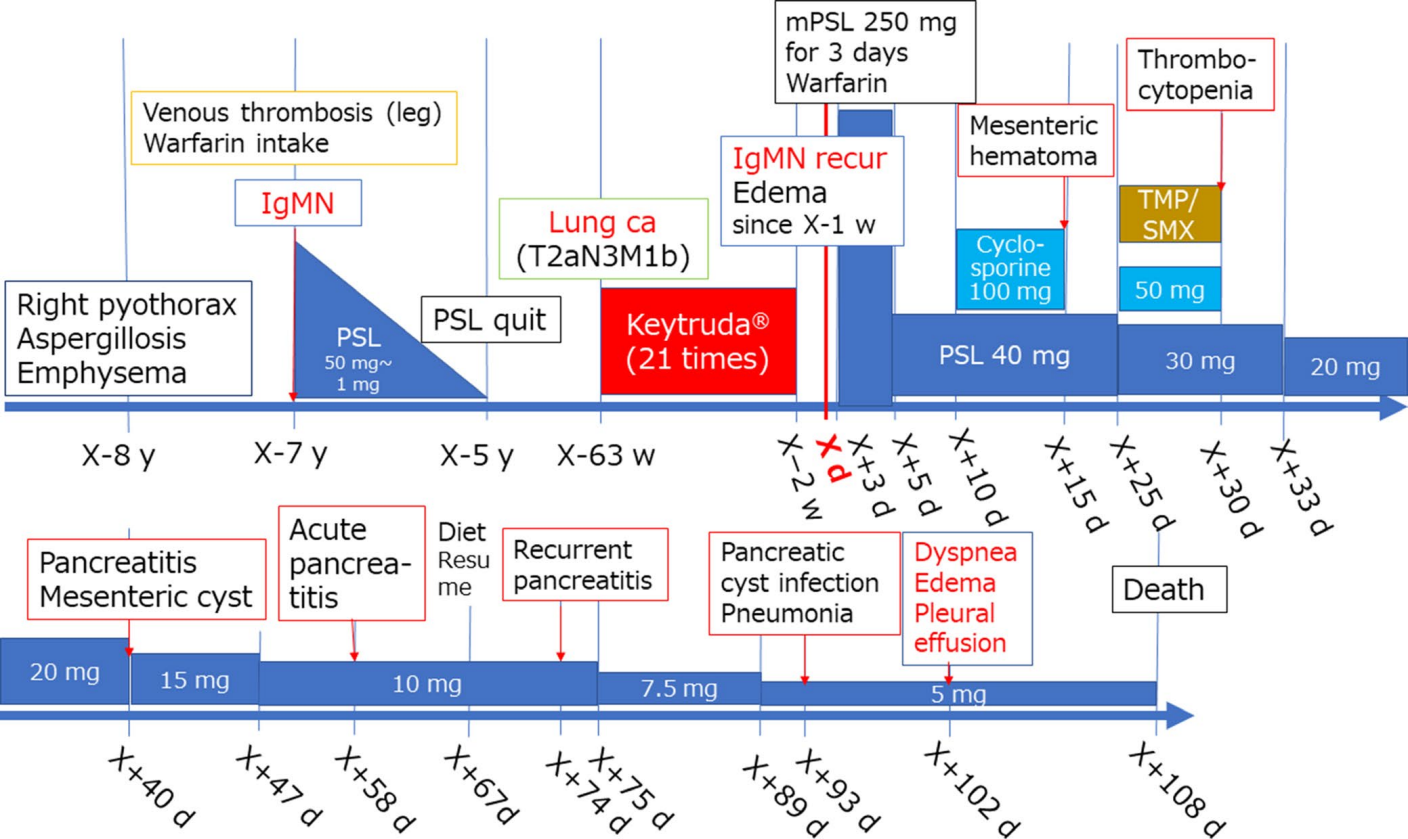
Schematic presentation of the clinical course of the male patient aged in his 60s

A schematic representation of the complex clinical course of the present patient is displayed in Figure 1. The date of hospitalization for relapsed IgM nephropathy was regarded as day X.

A Japanese man in his 60s had a history of right pyothorax, lung aspergillosis, and emphysema eight years earlier after he stopped smoking 30 cigarettes per day over the previous 40 years. One year later, he suffered deep venous thrombosis in both legs, and warfarin was administered.

**FIGURE 2:**
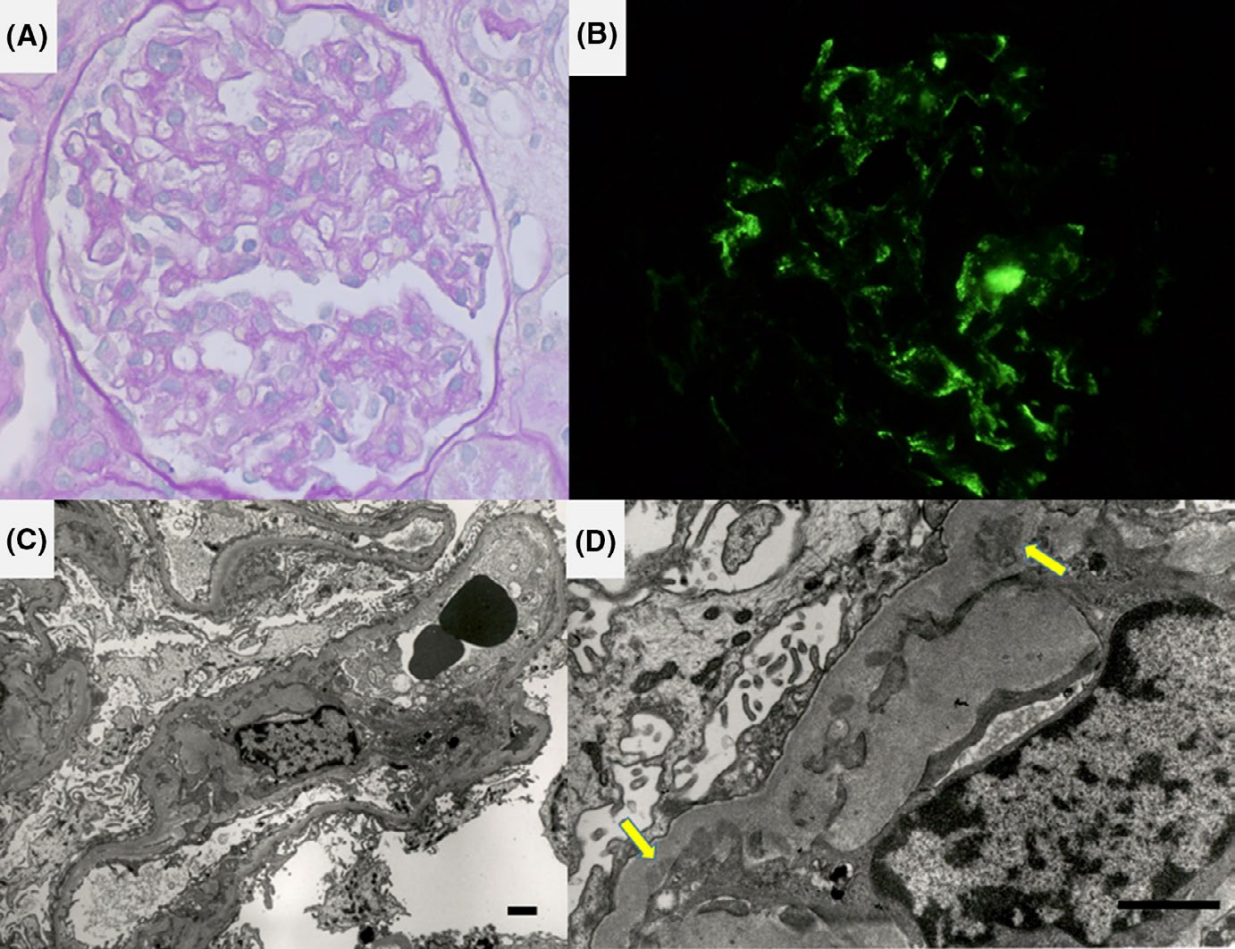
Microscopic appearance of renal biopsy specimen (A, periodic acid‐Schiff reaction, B, immunofluorescence study for IgM, C, D, electron micrographs, bars: 2 μm [C] and 1 μm [D]). The glomeruli resemble those of minimal change glomerulopathy with minimal increase of mesangial cells and matrix. Immunofluorescence study illustrates mesangial IgM deposition, and fine structural study shows foot process effacement and deposition of substances with low electron density in the mesangial matrix along the basement membrane (arrows)

Soon, peripheral edema worsened, and a diagnosis of nephrotic syndrome was made on the basis of urinary protein of 12 g/day, serum protein of 4.3 g/dL, and serum cholesterol of 330 mg/dL. Renal biopsy revealed IgM nephropathy with minimal mesangial cell growth and evident mesangial deposition of IgM. Electron microscopy revealed foot process effacement and mild increase of mesangial matrix with focal immune deposits having low electron density (Figure 2). Prednisolone therapy was started at 50 mg/d. Although the disease was steroid‐resistant, the steroid was gradually tapered to avoid exacerbation of pulmonary aspergillosis. Low‐density lipoprotein adsorption treatment was performed seven times, and proteinuria was improved as indicated by a urinary protein‐creatinine (Cr) ratio of 0.2 g/gCr. Two years after the onset of nephrotic syndrome, steroid therapy was completed with the patient in delayed remission.

Four years later, a 21‐mm adenocarcinoma of the lung developed in the peripheral part of the right upper lobe. Transbronchial lung biopsy disclosed the histological type as well‐differentiated papillary adenocarcinoma. Positron emission tomography‐computed tomography showed multiple metastases in both upper and lower lobes of the right lung, the hilar, mediastinal and supraclavicular lymph nodes, ribs, and pelvic and femoral bones (cT2aN3M1b). Molecular studies showed wild‐type epidermal growth factor receptor gene and negative anaplastic lymphoma‐kinase expression. The tumor proportion score of programmed cell death ligand‐1 was more than 95%. Pembrolizumab injection (200 mg) was started in week X‐63 and was continued every three weeks (for a total of 21 injections) until week X‐2, at which time the shadow images of the primary and metastatic tumor disappeared.

In week X‐1 (62 weeks after the initiation of the ICPI), peripheral edema reappeared, and on day X‐2, the urinary protein‐creatinine ratio had increased to 16 g/gCr. On day X, the patient was hospitalized with a diagnosis of relapsed nephrotic syndrome. Pembrolizumab was discontinued, and methylprednisolone 250 mg was administered for three days, followed by prednisolone 40 mg/day, with warfarin added to prevent thrombosis. Urinary proteinuria was unchanged, so a challenge with cyclosporine 100 mg/day was initiated on day X + 10. On day X + 15, the patient complained of abdominal pain, and computed tomography revealed a 9‐cm‐sized mesenteric hematoma. The patient's prothrombin time‐international normalized ratio was 3.17 (standard value: 1.5‐3.0). Prothrombin complex concentrate was administered, and the warfarin and cyclosporine were discontinued. The abdominal symptoms were gradually relieved, and on day X + 25, prednisolone was reduced to 30 mg/day and cyclosporine 50 mg was restarted together with the administration of sulfamethoxazole/trimethoprim (ST) compound. On day X + 30, thrombocytopenia (5.2 × 10^4^/mL) forced discontinuation of the cyclosporine and ST compound, after which the thrombocyte count soon recovered. On day X + 40, abdominal pain recurred, and an imaging study indicated enlargement of the mesenteric hematoma, which had become cystic and was 12 cm in size. On day X + 58, acute pancreatitis was diagnosed based on elevated serum levels of amylase at 538 U/L, AST at 406 U/L,ALT at 595 U/L, and swelling of the pancreas as revealed by computed tomography. The pancreatitis was controlled by diet fasting. After resumption of his diet on day X + 67, exacerbation of the pancreatitis recurred on day X + 74. His serum amylase level reached 1,600 U/L, and the mesenteric cystic lesion had shrunk to 6 cm in size.

Fever occurred on day X + 93, the mesenteric cyst had enlarged, and a chest shadow suggestive of interstitial pneumonia appeared. Infection of the mesenteric cyst was suspected, and a drainage tube was inserted. Because of the high level of amylase in the cyst fluid, a diagnosis of pancreatitis‐associated pseudocyst was made. On day X + 102, complications of peripheral edema, pleural effusion, and hypoxemia developed. Despite the injection of furosemide, hypoxia progressed, and the patient died of respiratory failure on day X + 108.

**FIGURE 3:**
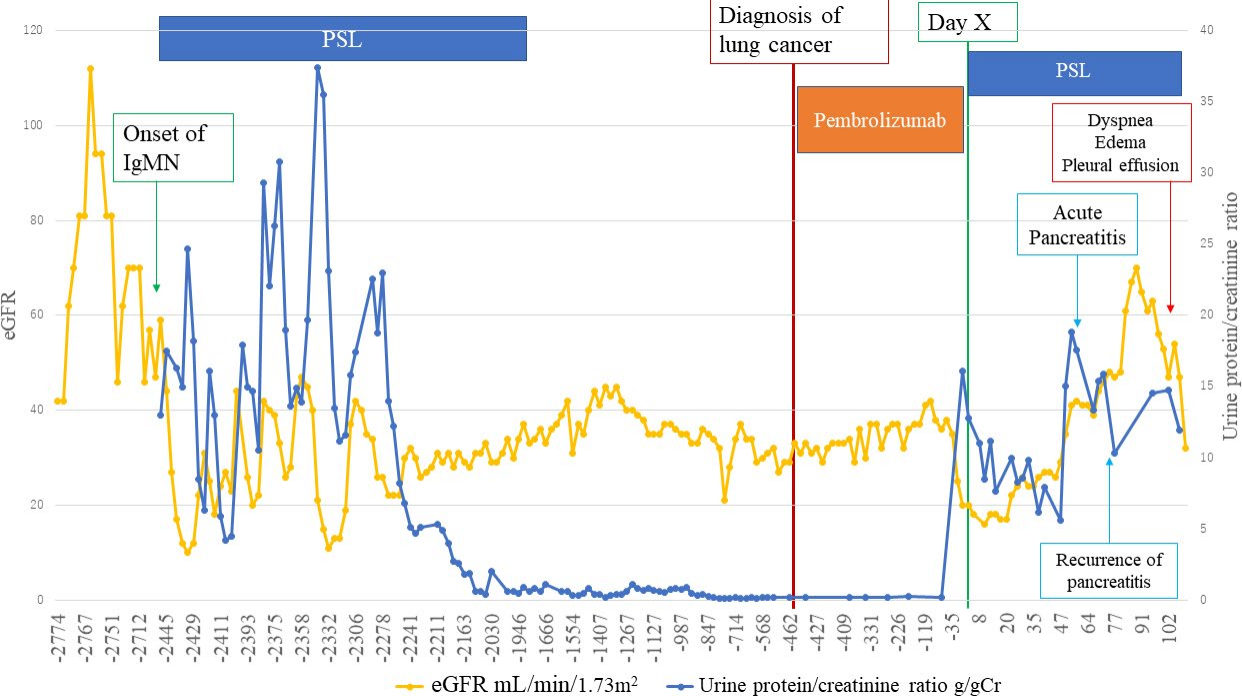
Profiles of changing renal functions throughout the patient's long clinical course. Two renal function indicators, eGFR (mL/min/1.73 m^2^), and urine protein‐creatinine ratio (g/gCr), are plotted. The key events are indicated by arrows

Figure 3 shows the profile of changing renal functions during the patient's long clinical course. Two types of renal function indicators, estimated glomerular filtration rate (eGFR) and urine protein g/g creatinine ratio (UP g/gCr), were chosen for the presentation. The initial prednisolone administration resulted in sustained suppression of proteinuria with moderate renal function impairment (eGFR around 30‐40) until day X.

## 3 AUTOPSY FINDINGS

Autopsy was performed one hour after death. The final autopsy diagnoses are summarized in Table 1.

**TABLE 1:** Final Autopsy Diagnosis (male aged 60s)

1. Lung cancer (right upper lobe, well‐differentiated adenocarcinoma, 9 mm in size)
a) S/P: administration of pembrolizumab (Keytruda^®^), 21 times
b) Metastasis: not identified (but with history of lung, nodal, and bone metastases)
2. IgM nephropathy (minimal change glomerulopathy with mesangial deposition of IgM)
a) Nephrotic syndrome (kidney weight: left 125 g, right 85 g)
b) Pleural effusion (left 1,800 mL, right 500 mL)
c) Edema in the lower extremities
3. Acute pancreatitis
a) Hemorrhagic cyst of the mesentery (7 cm in size)
b) Hemorrhagic cyst on the rectovesicular fossa
c) Fat necrosis in peripancreatic tissue
d) Mild pancreatic fibrosis
4. Usual interstitial pneumonia with acute exacerbation
a) Honeycomb lung (lung weight: left 280 g, right 465 g)
b) Hyaline membrane formation (diffuse alveolar damage)
5. Old pulmonary tuberculosis
a) Pleural fibrous adhesion, right
b) Encapsulated caseous focus, right middle lobe

**FIGURE 4:**
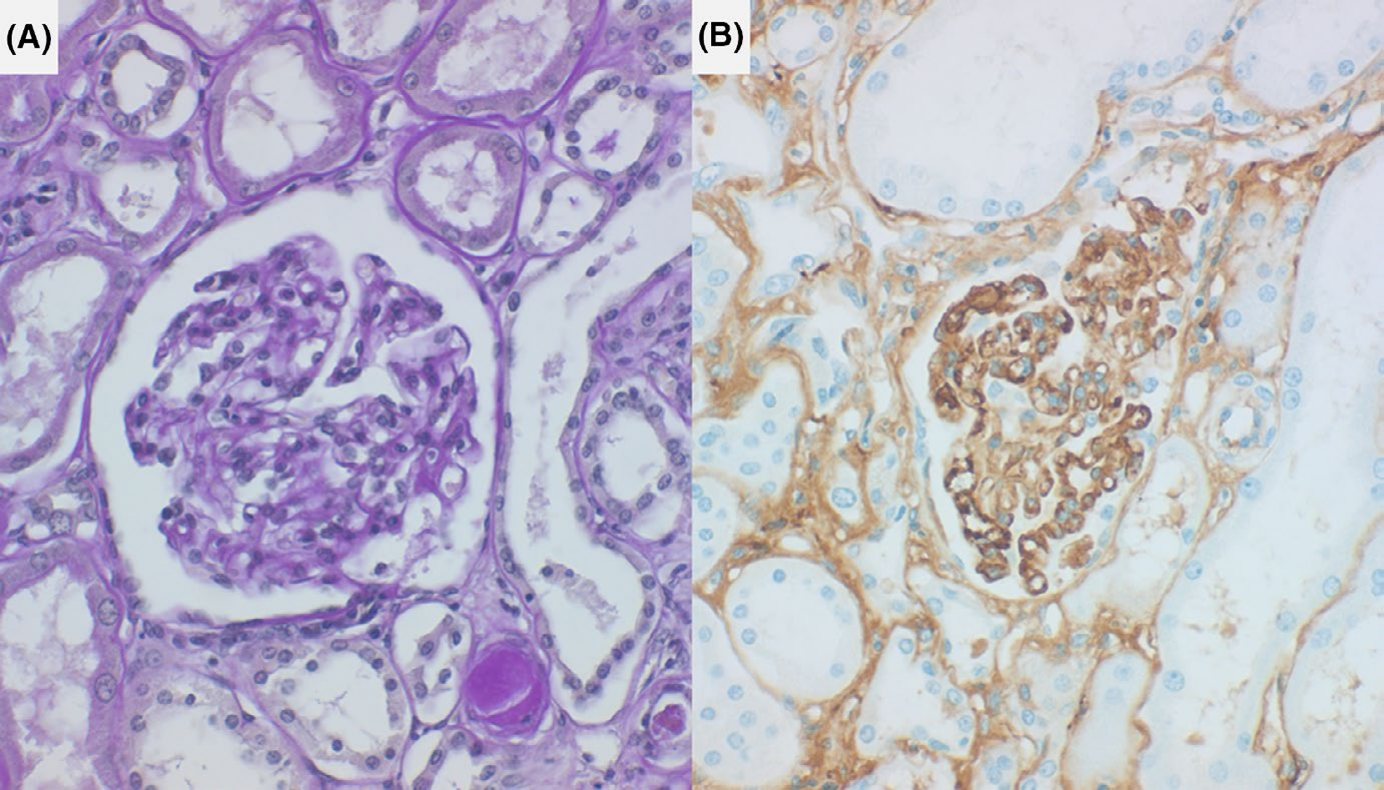
Microscopic features of autopsy kidney (left: periodic acid‐Schiff reaction, right: IgM immunostaining after prolonged protease‐1 digestion). Mesangial cell growth or increase of mesangial matrix is scarcely noted. Immunostaining after prolonged protease‐1 digestion of formalin‐fixed, paraffin‐embedded sections shows mesangial deposition of IgM

The kidneys were mildly atrophic and weighed 125 g (left) and 85 g (right). Microscopically, there were small subcapsular clusters of sclerotic glomeruli, but the remaining glomeruli scarcely showed mesangial cell growth or matrix increase. Interstitial fibrosis was mildly noted. After prolonged protease‐1 digestion of formalin‐fixed, paraffin‐embedded sections, ^8^, ^9^ mesangial deposition of IgM was shown, whereas, deposition of IgA and IgG was negative. These features were diagnostic of the relapse of IgM nephropathy (Figure 4).

**FIGURE 5:**
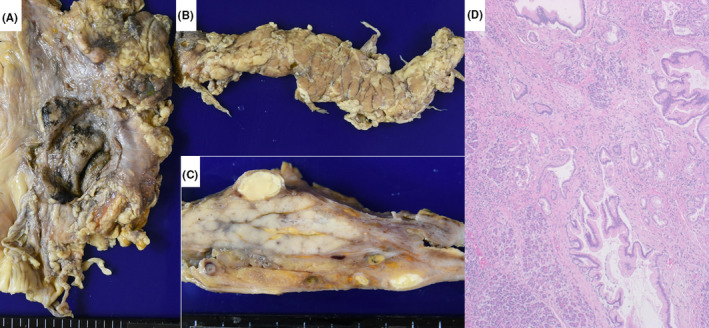
Gross appearance of mesenteric pseudocyst (a) and fat necrosis of peripancreatic tissue (b: overall appearance and c: cut surface) and microscopic features of the pancreas (d: HE staining). Old and hemorrhagic cyst of the mesentery, measuring 7 cm, is located adjacent to the uncinate part of the pancreas head. Fat necrosis is observed around the pancreas, and the cut surface displays encapsulated foci of fat necrosis. Mild interstitial fibrosis is discerned microscopically

An encapsulated, 7‐cm‐sized hemorrhagic mesenteric cyst with a drainage tube inserted was located just adjacent to the uncinate process of the pancreas head. Hemosiderin deposition was evident in the cyst wall. Encapsulated foci of fat necrosis were scattered in the peripancreatic fat tissue, and interlobular pancreatic septa were mildly fibrotic. Acute hemorrhagic necrosis of the pancreatic parenchyma was not observed. The mesenteric cyst was regarded as a pancreatitis‐associated lesion (pseudocyst). The pancreatic morphology is shown in Figure 5. A hemorrhagic cystic lesion was also distributed on the rectovesical peritoneal fossa (Douglas’ fossa).

**FIGURE 6:**
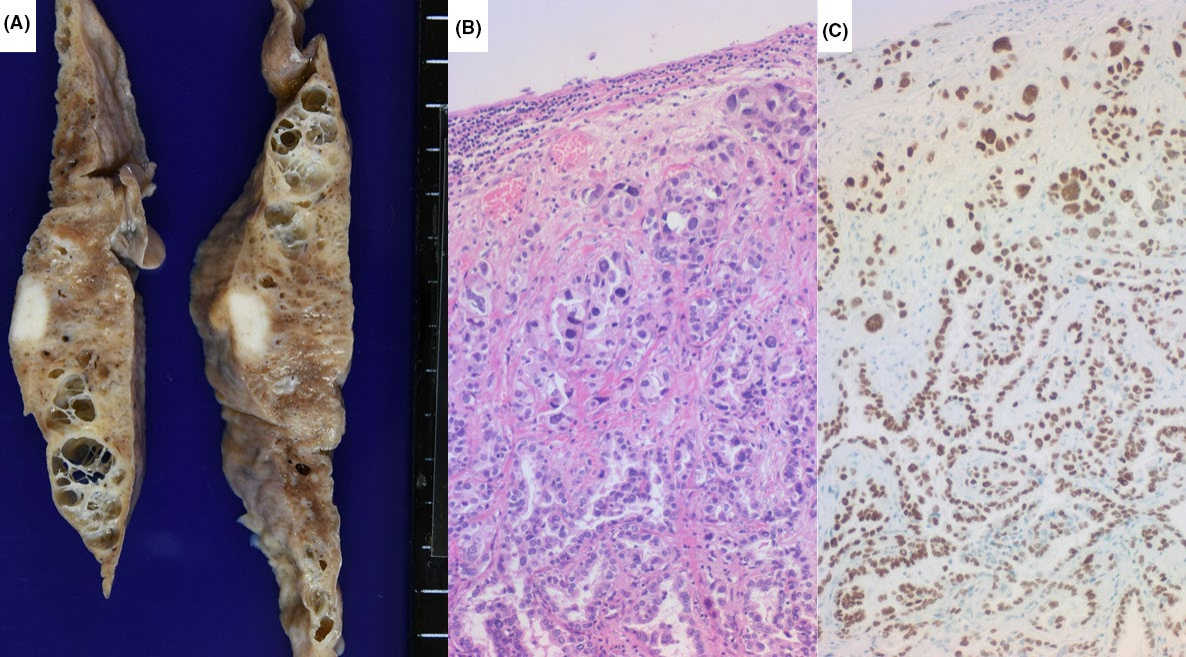
Pulmonary adenocarcinoma in fibrotic lung parenchyma (a: gross appearance of right upper lobe, b: HE staining, c: thyroid transcription factor‐1 immunostaining). The 9‐mm white nodule is located just beneath the pleura. The surrounding lung tissue is fibrotic and honeycombed, focally resembling emphysema. Microscopically, well‐differentiated papillary adenocarcinoma shows neither necrosis nor lymphocytic infiltration. The nuclei of the cancer cells are diffusely immunoreactive for thyroid transcription factor‐1

A 9‐mm‐sized, white‐colored subpleural lung nodule was noted in the anterior part of the right upper lobe. Microscopically, well‐differentiated adenocarcinoma of papillary type was predominant, but focally with a component of poorly differentiated adenocarcinoma. Neither necrotic change nor lymphocytic infiltration was observed. The cancer cells were immunoreactive for thyroid transcription factor‐1, napsin A, and cytokeratin 7, but negative for cytokeratin 5/6 (Figure 6). Metastatic deposits were not confirmed in other organs and tissues.

**FIGURE 7:**
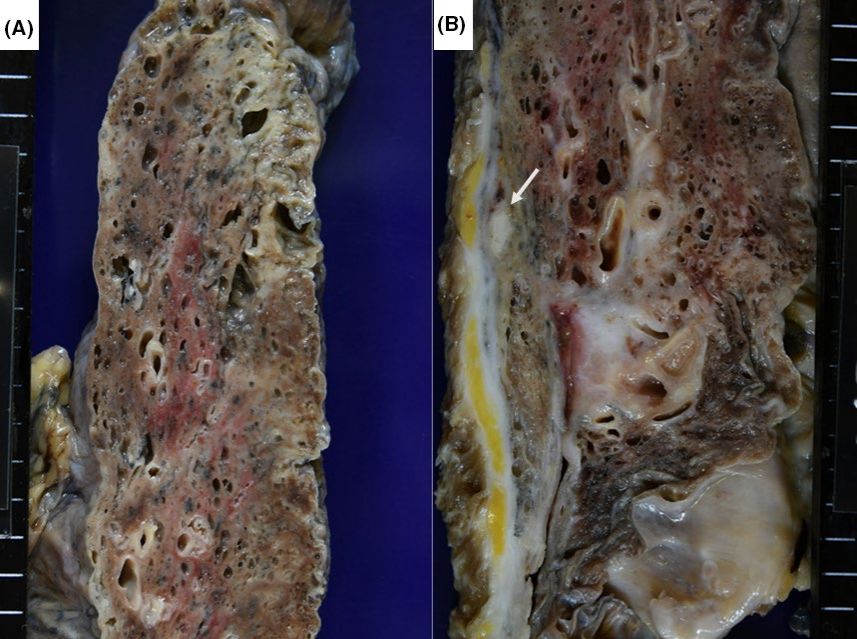
Gross appearance of usual interstitial pneumonia (pulmonary fibrosis) with acute exacerbation (a: left upper lobe, b: right upper and middle lobes). Focal subpleural honeycombing and infiltrative change in the lung parenchyma are noted. The right pleura shows diffuse fibrous adhesion, and an encapsulated caseous focus is noted beneath the pleura of the collapsed middle lobe (arrow)

**FIGURE 8:**
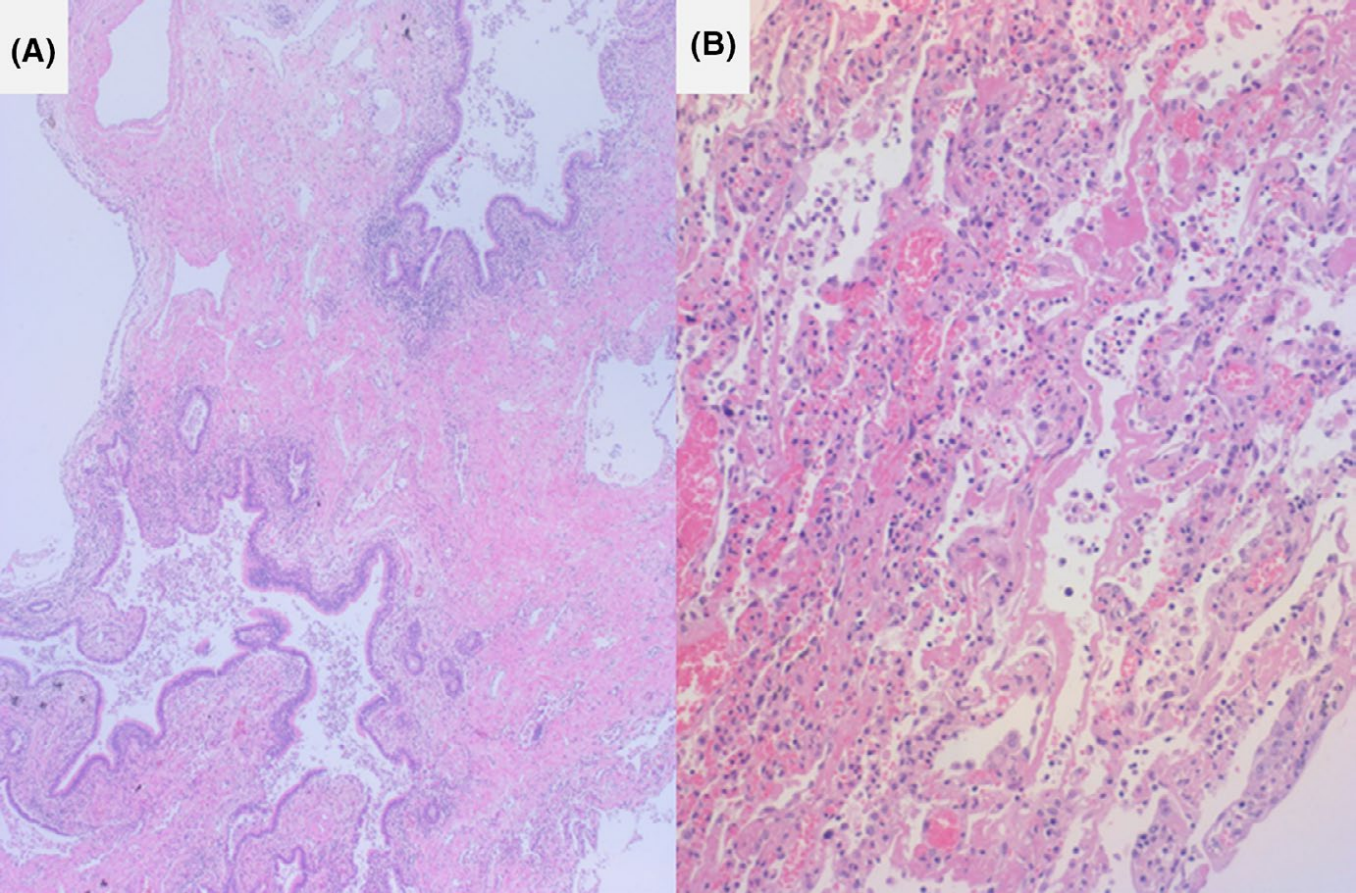
Microscopic features of usual interstitial pneumonia (pulmonary fibrosis) with acute exacerbation (HE staining, a: honeycomb lung, b: hyaline membrane formation). Interstitial fibrosis with disappearance of normal alveolar structures is observed. Hyaline membrane formation is noted at the site of acute exacerbation

Gross examination of the non‐neoplastic lung revealed subpleural honeycombing and multifocal parenchymal infiltrative change bilaterally (Figure 7). The lungs weighed 280 g (left) and 465 g (right). Microscopically, the pre‐existing alveolar structures were often distorted and surrounded by interstitial fibrosis, and were associated with features of diffuse alveolar damage with hyaline membrane formation (Figure 8). These features were consistent with acute exacerbation of usual interstitial pneumonia. The right pleura showed severe fibrous adhesion in association with a small encapsulated caseous nodule in the collapsed right middle lobe. The pleural lesion was thus regarded as old tuberculosis (see Figure 7). The patient had a history of pulmonary aspergillosis, but *Aspergillus* infection was not identified.

## 4 DISCUSSION

The disease entity of IgM nephropathy is controversial. IgM nephropathy may be a variant of minimal change nephrotic syndrome or focal segmental glomerulosclerosis, but it occurs commonly in adults and is often steroid‐resistant. ^5^, ^6^, ^7^ The present patient underwent two years of steroid administration before achieving delayed remission, and a late relapse of nephrotic syndrome occurred after repeated use of an ICPI. Mesangial IgM deposition was proven by prolonged protease digestion of the formalin‐fixed, paraffin‐embedded autopsy kidney. Antibody molecules can bind cryptic antigens after the rigorous protease digestion step, because formalin‐mediated cross‐linkage between protein networks in the tissue is significantly loosened, as has been reported previously. ^8^, ^9^

Administration of ICPIs may provoke irAEs in a variety of organs and tissues. Early detection of irAEs and discontinuance of ICPIs are required in clinical practice. ^10^ Renal irAEs are infrequent at around 4%: glomerulonephritis is especially rare, but interstitial nephritis is relatively common. ^11^ The period from the initiation of ICPI therapies to acute renal injury ranges from 21 to 245 days (median 91 days), whereas that from its cessation to acute renal injury ranges from 7 to 63 days (median 21 days). ^12^ In the present case, IgM nephropathy relapsed 63 weeks after the initiation of ICPIs, as indicated in Figure 3. Such a late‐occurring irAE is quite unusual.

ICPIs stimulate T‐lymphocytes to battle cancer cells. It is thus reasonable to suppose that the immune activation by ICPIs provoked the relapse of IgM nephropathy, although the detailed mechanism remains unclear. In patients with immune‐related disorder, 75% were accompanied by relapse/exacerbation of the disease after administration of ICPIs but with little evidence for inducing novel‐type irAEs. ^3^ In cases of malignant melanoma complicated by immune‐related disorders, the anti‐tumor effect of ICPIs was much more beneficial than the risk of relapse/exacerbation of the immune‐related disorders. ^13^ In patients with nonsmall cell lung cancer complicated by immune‐related disorders, 55% exhibited exacerbation of the disorders, but 74% of the irAEs were regarded as being controllable at grades 1‐2. ^14^ Immunosuppressants including steroid administered to control the immune‐related disorder did not influence the anti‐tumor effect of ICPIs. ^15^

The irAEs tended to occur when the volume of ICPI therapy was increased, ^16^ and they happened within 12 weeks after the initiation of the therapy. ^2^ In the present case, after the late relapse of IgM nephropathy, mesenteric hematoma (hemorrhagic pseudocyst) occurred on day X + 15 and exacerbated on days X + 40 and X + 93, and the diagnosis of acute pancreatitis was made on day X + 58. The complication of acute exacerbation of pulmonary fibrosis developed on day X + 93, two weeks prior to the patient's death. Acute pancreatitis was histopathologically proven at autopsy by the presence of hemorrhagic pseudocysts in the mesentery and Douglas’ fossa, multifocal fat necrosis, and mild interstitial fibrosis of the pancreas. In the lung, diffuse alveolar damage with hyaline membrane formation was recognized among honeycombing pulmonary fibrosis of usual interstitial pneumonia type.

Whether the pancreatic and pulmonary complications in the present case were immune‐related could not be definitely confirmed, but the possibility of ICPI‐associated irAEs was strongly suspected. Acute pancreatitis is a rare complication of ICPI‐associated irAEs. In a previous study, increases in the serum levels of amylase and lipase were recorded in only two of 119 cases evaluated, but no associated symptomatic pancreatic lesions were present. ^17^ Acute fibrinous and organizing pneumonia and diffuse alveolar damage are listed as ICPI‐related irAEs of the lung, and they are often lethal. ^18^, ^19^ In fact, ICPI‐related lung lesions were encountered more often in patients with nonsmall cell lung carcinoma than in those who suffered from other types of malignancy.^20^, ^21^ The association of the background IgM nephropathy is supposed to be an important factor for the pulmonary irAEs, and further study and the accumulation of similar cases are needed for clarifying their pathophysiology and pathogenesis.

The remote effects of ICPIs are debatable, and the recognition or definition of irAEs may be difficult. However, the present case indicates that clinicians should be aware of the possibility of late complications of ICPI administration.

## 5 CONCLUSION

Administration of ICPIs to cancer patients with immune‐related disorders has not yet been thoroughly investigated. In the present case, an ICPI was adequately effective against primary and metastatic foci of adenocarcinoma of the lung. However, repeated use of the ICPI provoked a late relapse of IgM nephropathy along with late, intractable, and lethal complications in the pancreas and lung. Accumulation of similar cases will be necessary to determine the appropriate use of ICPIs in patients with immune‐related disorders.

## CONFLICT OF INTEREST

The authors do not have any conflicts of interest to declare in relation to the present report. There were no sources of funding for reporting the present case.

## AUTHOR CONTRIBUTIONS

We declare that all the authors made a substantial contribution to the concept of the case report or interpretation of data and approved the version to be submitted. Each author has participated sufficiently in the work to take public responsibility for appropriate portions of the content.

## STATEMENT OF ETHICS

The patient was unmarried, so his intimate co‐worker provided written informed consent for the publication of this case report. The study was conducted ethically in accordance with the World Medical Association Declaration of Helsinki.

## Data Availability

The datasets generated and/or analyzed during the current report are available from the corresponding author on reasonable request.
